# Designing an Internationally Accessible Web-Based Questionnaire to Discover Risk Factors for Amyotrophic Lateral Sclerosis: A Case-Control Study

**DOI:** 10.2196/resprot.4840

**Published:** 2015-08-03

**Authors:** Jane Alana Parkin Kullmann, Susan Hayes, Min-Xia Wang, Roger Pamphlett

**Affiliations:** ^1^ The University of Sydney Camperdown NSW Australia

**Keywords:** amyotrophic lateral sclerosis (ALS), motor neuron disease (MND), web-based, online, questionnaire, epidemiology, risk factor, case-control study, international, language translation

## Abstract

**Background:**

Amyotrophic lateral sclerosis (ALS) is a progressive neurodegenerative disease with a typical survival of three to five years. Epidemiological studies using paper-based questionnaires in individual countries or continents have failed to find widely accepted risk factors for the disease. The advantages of online versus paper-based questionnaires have been extensively reviewed, but few online epidemiological studies into human neurodegenerative diseases have so far been undertaken.

**Objective:**

To design a Web-based questionnaire to identify environmental risk factors for ALS and enable international comparisons of these risk factors.

**Methods:**

A Web-based epidemiological questionnaire for ALS has been developed based on experience gained from administering a previous continent-wide paper-based questionnaire for this disease. New and modified questions have been added from our previous paper-based questionnaire, from literature searches, and from validated ALS questionnaires supplied by other investigators. New criteria to allow the separation of familial and sporadic ALS cases have been included. The questionnaire addresses many risk factors that have already been proposed for ALS, as well as a number that have not yet been rigorously examined. To encourage participation, responses are collected anonymously and no personally identifiable information is requested. The survey is being translated into a number of languages which will allow many people around the world to read and answer it in their own language.

**Results:**

After the questionnaire had been online for 4 months, it had 379 respondents compared to only 46 respondents for the same initial period using a paper-based questionnaire. The average age of the first 379 web questionnaire respondents was 54 years compared to the average age of 60 years for the first 379 paper questionnaire respondents. The questionnaire is soon to be promoted in a number of countries through ALS associations and disease registries.

**Conclusions:**

Web-based questionnaires are a time- and resource-efficient method for performing large epidemiological studies of neurodegenerative diseases such as ALS. The ability to compare risk factors between different countries using the same analysis tool will be of particular value for finding robust risk factors that underlie ALS.

## Introduction

Amyotrophic lateral sclerosis (ALS, also known as motor neuron disease or MND) is a progressive neurodegenerative disease of adults with a usual survival of three to five years after diagnosis [1]. Epidemiological studies using traditional methods of collecting data via mailed paper questionnaires or via telephonic or in-person interviews have so far not revealed any widely accepted environmental or lifestyle risk factors for ALS.

Previous epidemiological studies of ALS have had a number of limitations. ALS has an incidence of about 2-3 per 100,000 in most populations, so it is not a common disorder and obtaining large numbers of respondents has been difficult [2]. No intercountry comparisons of risk factors for ALS using the same survey tool have been undertaken. Restricting the geographical region of recruitment to one country or continent prevents identification of risk factors that vary across countries [3] or ethnic groups. As new criteria to classify ALS into its sporadic and familial forms are proposed, changing diagnostic criteria will make characterisation of cases in previous studies difficult [4]. In addition, new potential environmental risk factors for ALS are continually being proposed, but it is inconvenient to add questions to non-Web surveys.

We became aware of these and other limitations of paper-based questionnaires during the course of an Australian study looking for risk factors for sporadic ALS. Despite this being a continent-wide survey undertaken over 11 years (2000-2011) with active recruitment of participants by state-based ALS associations, responses were obtained from only 812 ALS patients and 793 nonrelated controls in a population of 23 million people. Although this remains one of the largest epidemiological case-control databases in ALS with several publications arising from the study [2,3,5-10], numbers were too small to analyse subgroups in many categories, such as those for less common occupations. The majority of respondents were English-speaking and of western European descent although people from many language groups live in Australia (some of this bias can be explained by the questionnaire being available only in English). The criteria we used for separating familial and sporadic ALS are under revision, and many of the patients we classified as having familial ALS would now be considered to be in the sporadic group [4]. The financial cost to obtain and process information was high and when funding for staff and consumables came to an end, the survey had to close. We did not ask questions about topics such as psychiatric conditions since the respondents had to identify themselves, and this understandably would have made many reluctant to give out such information.

Many of the limitations experienced during our paper-based study have been overcome by migrating to an online questionnaire, where respondents are not asked for personally identifiable information. Our Web-based questionnaire can be easily translated into other languages for both reading and answering questions, which will aid recruitment and allow for international comparisons of risk factors. We describe our approach to designing this online questionnaire to look for risk factors in ALS and present the initial responses to this survey. We also summarise the advantages and disadvantages of Web-based versus paper-based questionnaires as they pertain to looking for risk factors for neurodegenerative diseases.

## Methods

###  Questionnaire Software

Questionnaire platforms from a number of providers were evaluated as potential sources of Web-based survey software. Most offered user-friendly survey design, secure storage of respondent data, an online log-in portal that allows users to access the survey from any Internet browser, and the ability to download survey data in several formats. Qualtrics [11] was identified as a good platform for our project because it is flexible and provides a large diversity of question types.

### Questionnaire Design

#### Overview

Relevant questions from our original paper-based ALS questionnaire were entered into the Qualtrics platform using the appropriate question formats (see Multimedia Appendix 1 to view the original paper-based questionnaire). The design of our online questionnaire was based on recent recommendations of best practice in this field [12-14].

#### Pay Careful Attention to the Wording of Questions to Ensure Clarity

Our experience with our previous paper-based questionnaire was helpful in identifying types of questions that tended to result in ambiguous answers.

#### Use Predetermined Choices to Ensure Standard Answers

For example, questions requiring a written answer in a paper-based questionnaire (eg, “In which country are you currently living?”) can be formatted as a single-choice drop-down menu in a Web-based format. The number of answers requiring text entry, which can cause transcription difficulties and delay access to the data, was reduced to a minimum in the online questionnaire.

#### Place Questions Into Topic Groups

The online questionnaire is organised according to topics of interest (eg, occupation, exercise). This improves coherence of the questionnaire, and it also allows easier topics to be placed towards the beginning of the survey to increase respondents’ confidence about entering data into the questionnaire.

#### Use Automated Question Logic

Question logic shows or skips certain questions based on previous answers. This relieves respondents of the responsibility of following the logic of a paper-based questionnaire, and ensures they only need view questions that apply to them. Question logic largely eliminates commission errors (ie, answering questions that are not applicable) and omission errors (ie, not answering questions that are applicable) [14]. Question logic applies to about 25% of our online questions.

#### Avoid Use of a Progress Bar

A progress bar, which shows respondents how far into the survey they are, was not used. First, a progress bar would have been misleading because it does not take into account the show/skip logic within the questionnaire. Second, a progress bar is not recommended on longer surveys because it discourages completion [14].

### Access for Patients With Physical Disabilities

Access to the questionnaire was a concern given that respondents with ALS could have limited mobility. We therefore ensured the questionnaire is compatible with speech-to-text programs and spoken commands. To aid visibility, we set the default font size at 12 point, made the text of all questions in bold font, and implemented a software feature that highlights the question being worked on.

### Access in Different Languages

We plan to translate the questionnaire into many languages, including all languages spoken in countries within the International Alliance of ALS/MND Associations. Respondents will select their preferred language from a list of available translations before entering the questionnaire. For text entry, respondents will be able enter answers in their own language. Since only a few questions are answered by entering text, translations to English will not be onerous.

Google Translate is used to perform the first rough translation of non-English languages, but fluent speakers of both English and the language to be translated need to spend many hours amending this to obtain the correct meaning and grammar in the text, based on the English version. For example, in our question about skin color, the word *fair* in most languages is translated as *reasonable* rather than the intended meaning of *light in color*. Qualtrics has a function in which the English and Google-translated non-English version of the questionnaire can be presented side-by-side, so the translator can readily edit the non-English version with reference to the meaning in the English version.

We have chosen first to check and adjust the translation of simplified Chinese, one of the languages where Google Translate appears to give the greatest number of ambiguities. Fluent speakers of other languages are in the process of checking other Google translations. The Google-translated languages that have been checked for accuracy (only simplified Chinese at the time of manuscript submission) will be indicated in the language list as available translations.

### New Content in the Web-Based Questionnaire

#### General

The content of our paper-based questionnaire was compared to the Stanford University ALS Consortium of Epidemiologic Studies (ACES) questionnaire [15], and questions were added or modified on topics such as alcohol and tobacco use, medical history, hobbies and pastimes, and pesticide and chemical exposures. The differences in our paper- and Web-based questions can be viewed by comparing the paper-based questionnaire in Multimedia Appendix 1 and the online questionnaire [16].

#### Defining Familial Versus Sporadic ALS

Controversy persists as to the definition of familial versus sporadic (or isolated) ALS, with some clinicians classifying a patient as having familial ALS only if close family members also have the disease [4]. Based on studies of the heritability of familial ALS, the questionnaire now asks for the number of first-, second-, and third-degree relatives as well as more distant relatives who have ALS [4,17,18]. It further asks for the total number of first-, second-, and third-degree relatives in the respondent’s family overall, since the familial nature of a disease is harder to detect in a small family. Having this detailed family history will allow researchers who have access to our survey data to use their own criteria to define familial and sporadic ALS.

#### Dementia

Questions are now asked about the number of family members diagnosed with frontotemporal dementia (FTD), a recently recognised component of an ALS/FTD disease continuum [19]. This will allow our study to identify families where one member has ALS while another has FTD.

#### Genetic Variants

We now ask whether any ALS patient or relative has been identified as having a genetic variant associated with ALS. We do not ask respondents to identify the particular genetic variant since rare variants could constitute personally identifiable information.

#### ALS Functional Status

People with ALS are asked to complete the ALS Functional Rating Scale (ALS-FRS) [20] to assess their physical state at the time of taking the questionnaire. This will allow an assessment of the rate of progression of the disease, which can be calculated from the time of disease onset. A Web-based format for the ALS-FRS has previously been validated by comparing Web and in-person evaluations [21].

#### Physical Activity

To evaluate physical activity, which has been suggested to be a risk factor for ALS [22], questions were obtained from surveys used by the European Amyotrophic Lateral Sclerosis Consortium (EURALS) [23] and the European Multidisciplinary ALS Network Identification to Cure Motor Neuron Degeneration (Euro-MOTOR) [22].

#### Ratio of Finger Lengths

**Figure 1:**
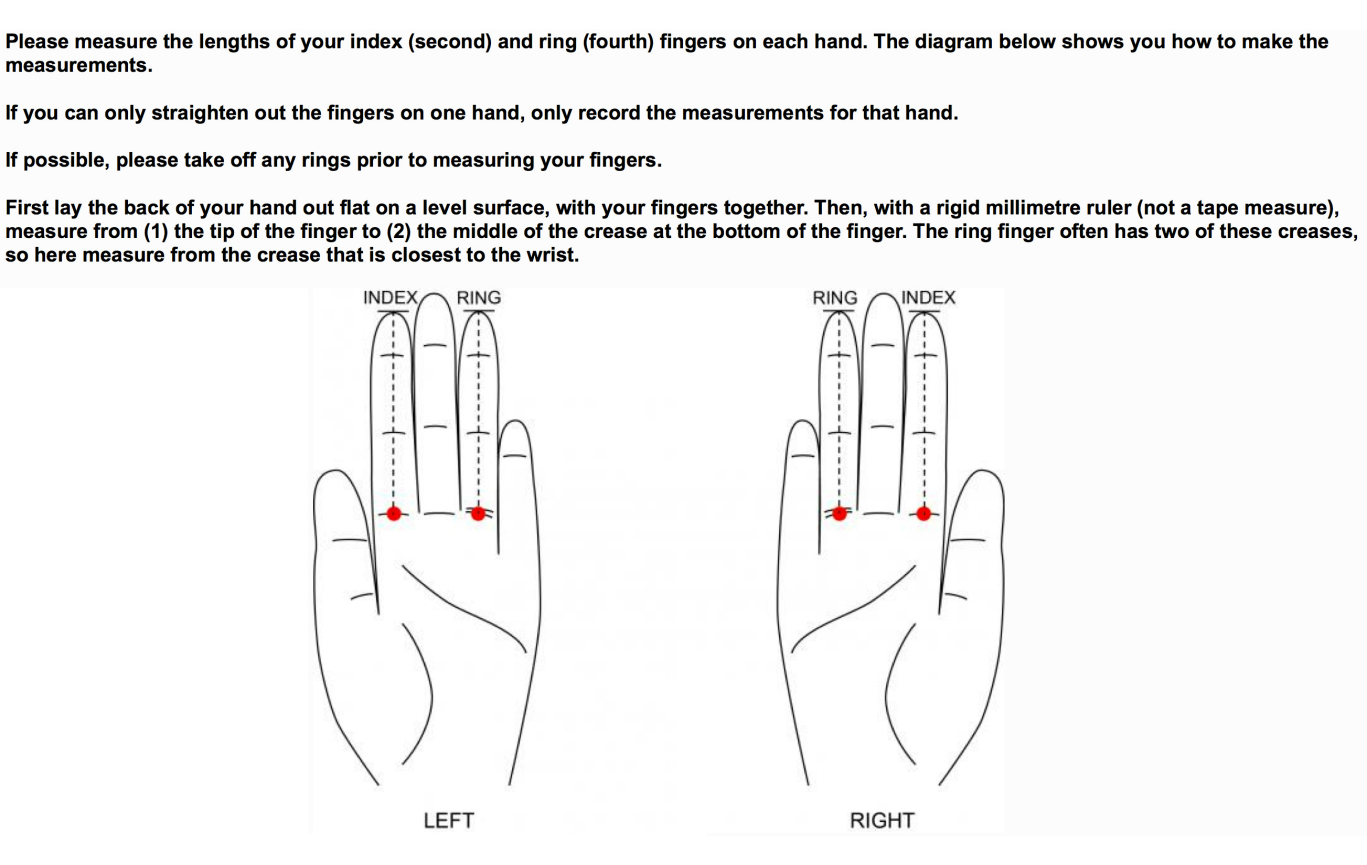
This diagram in the questionnaire shows respondents how to measure the length of their index and ring fingers. Below the question, boxes are provided (not shown) for respondents to select the measurements from a drop-down menu.

The ratio between the length of the ring finger and index finger, associated with prenatal exposure to testosterone, has been implicated as a risk factor for ALS [24]. A diagram has been included to show respondents how to perform and report these measurements (Figure 1). The reliability of these self-reported finger measurements is currently being investigated by photographing 100 volunteers’ hands and comparing their own finger measurements with measurements by researchers using the photographs.

#### Male Pattern Baldness

It has been suggested that men with early-onset alopecia have a higher risk of ALS [25]. We therefore included a question used by the Physicians’ Health Study in which men estimate the pattern of any hair loss they may have had when they were 45 years old by selecting one of 5 images ranging from no to marked hair loss [26,27].

#### Head Trauma

Head trauma has been implicated as a risk factor for ALS [28]. To gauge a history of head trauma, we added questions from the Retrospective Screening of Traumatic Brain Injury (RESTBI) Questionnaire [29].

#### Sun Exposure

Vitamin D deficiency has been implicated as a factor in ALS [30]. In most countries, sun exposure is the main source of vitamin D [31], but assessing sunlight exposure over long periods of time with a questionnaire is difficult [32]. We therefore asked about two aspects of sun-induced vitamin D generation, skin color and the reaction of the skin to sunlight, as used in the NSW Prostate Cancer Care and Outcomes Study [33].

#### L-BMAA

Because of the interest in a possible connection between the environmental toxin β-N-methylamino-L-alanine (L-BMAA) and ALS, we included questions related to L-BMAA exposure based on the French BMAALS program questionnaire [34].

#### Stress

Stress has been suggested as a potential risk factor for ALS [35]. Our questionnaire asks systematic questions about stress as a risk factor for the disease. To assess lifetime stress we used the Social Readjustment Rating Scale which scores the stress associated with a variety of events [36]. To evaluate the likely impact these stressors would have had on respondents, we used the Big Five personality traits assessment [37-39], the Connor-Davidson Resilience Scale [40], and the Geriatric Anxiety Inventory [41,42]. A Web-based administration of a scale similar to the Geriatric Anxiety Inventory has been validated by comparing Web and telephone interview surveys [43].

#### Diagnosis of ALS

On our previous paper-based questionnaire, we asked neurologists of ALS patients to send us copies of their clinical notes so that the type of ALS the respondent had could be assessed (there are four major types of the disease). This required a consent form specific to Australia, and individual neurologists around the country had to be contacted. No response was received from neurologists for about 15% of respondents, whose questionnaire data could not then be used. Since we designed the current questionnaire to be used for international comparisons of ALS risk factors, a direct approach to neurologists in different countries was not ethically feasible. On the online questionnaire, we therefore ask ALS patients to choose which type of ALS they have been diagnosed with from a predetermined list, and ask them to contact their neurologist or family doctor if they are unsure about the type.

### Avoidance of Culturally Specific Questions

All questions were checked for content that could cause misunderstandings in different countries and cultures. We avoided questions that relate specifically to cultural or environmental aspects of any country.

### Information for Participants

Text providing information for participants (administrative details about the questionnaire), comprehensive instructions (how to complete the questionnaire), and guidelines (tips for using the questionnaire) appear after respondents access the questionnaire. Respondents then need to answer a few questions before being able to fully access the questionnaire. Respondents are asked to select age, gender, and whether they have ALS. They are asked to describe their connection to ALS if they do not have the disease. If they have a friend or partner with ALS, respondents are asked to list the length of the relationship. Last, respondents are asked how they heard about the questionnaire. After these are answered, an online consent form is displayed; once this is completed, respondents enter the main body of the questionnaire. All other questions are voluntary, but if a question is not able to be answered there is usually an option to explain why (eg, not applicable).

### Pairing of Cases and Matched Controls

ALS patients are asked to nominate (if available) a spouse/partner and friends to complete the questionnaire. ALS patients create a unique code and provide it to their spouse/partner and friends. The code is then used to link the ALS patient to these matched controls. This enables paired statistics to be performed on people who are likely to have similar environmental exposures; these statistics will be used for comparisons with nonmatched controls. The code does not allow participants to view other responses.

### Questionnaire Distribution

Qualtrics provides two means by which a questionnaire may be distributed: via an anonymous link or via an email invitation with a link specific to each respondent. We chose the anonymous option to maintain participant confidentiality. The questionnaire does not ask for any personally identifiable information such as name, email address, employer name, or exact locations lived. This preserves the anonymity of respondents, which is important considering the sensitivity of some of the data (eg, psychiatric history) being collected. In addition, the anonymous option allows distribution of the questionnaire to a wide international group of potential respondents.

### Recruitment of Participants

People both with and without ALS are being sought to complete the questionnaire. The only exclusion criterion is being under the age of 18 years, so there is little possibility for confusion about eligibility criteria. ALS patients in Australia are recruited via newsletters, Facebook pages, and meetings of ALS associations in each state. Nonmatched controls are recruited in particular among community groups such as Rotary International. In the United States, participants are recruited through the government-funded National ALS Registry at the Agency for Toxic Substances and Disease Registry (Centers for Disease Control and Prevention), which has been used by other researchers to recruit participants for ALS online epidemiological surveys [44]. Participants in other countries will be recruited through their respective national ALS associations with the assistance of the International Alliance of ALS/MND Associations.

### Data Collection and Storage

Responses to the questionnaire are initially placed on password-protected Qualtrics servers in the countries that host these servers. The Qualtrics servers in the United States are used in countries that do not have their own Qualtrics servers. Completed questionnaire responses are downloaded and transferred from the Qualtrics server into Excel (Microsoft Corporation) and SPSS (IBM Corporation) program files on a regular basis. The original responses are deleted from the Qualtrics servers every six months. Questionnaire responses are kept in a password-protected file on a password-protected computer at the University of Sydney. This computer is connected to Wi-Fi only via password-protected networks.

## Results

### Cases and Controls

Major groups in the study comprise those who have been diagnosed by a neurologist as having ALS (cases), spouse/partners and friends of people with ALS (matched nonrelated controls), blood relatives of people with ALS who do not have the disease (matched related controls), and persons completing the survey who do not fall into the other categories (nonmatched controls).

### The Online Questionnaire

**Figure 2:**
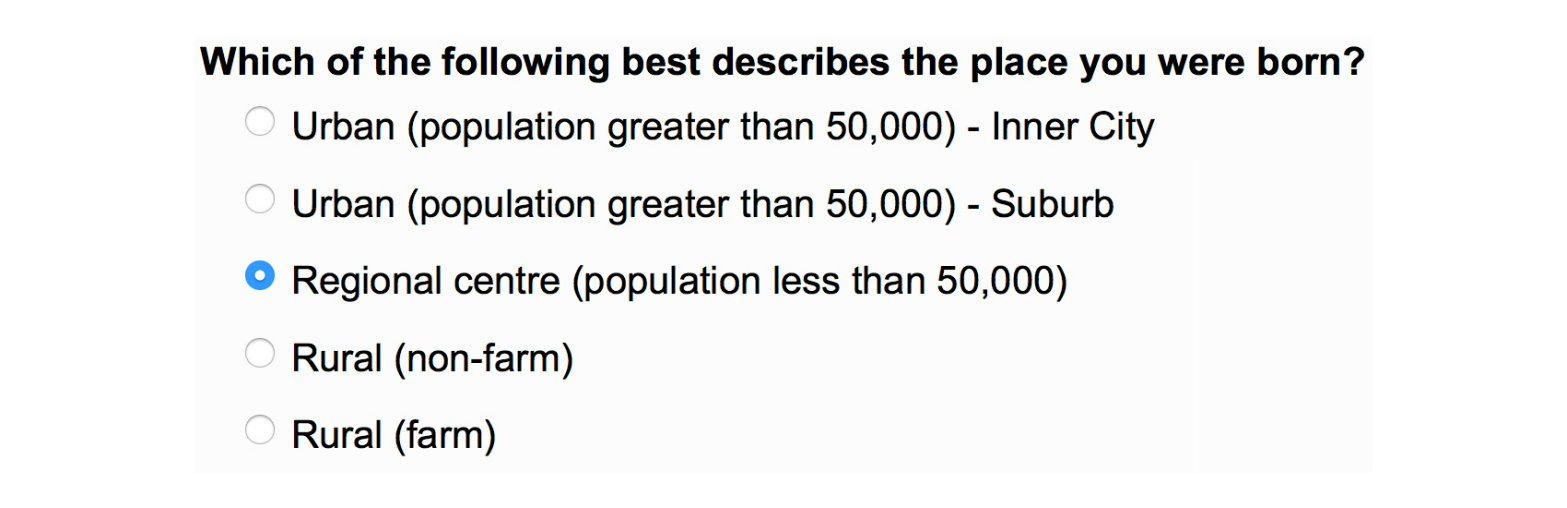
Example of a single-choice question. Only one choice of place of birth is allowed.

**Figure 3:**
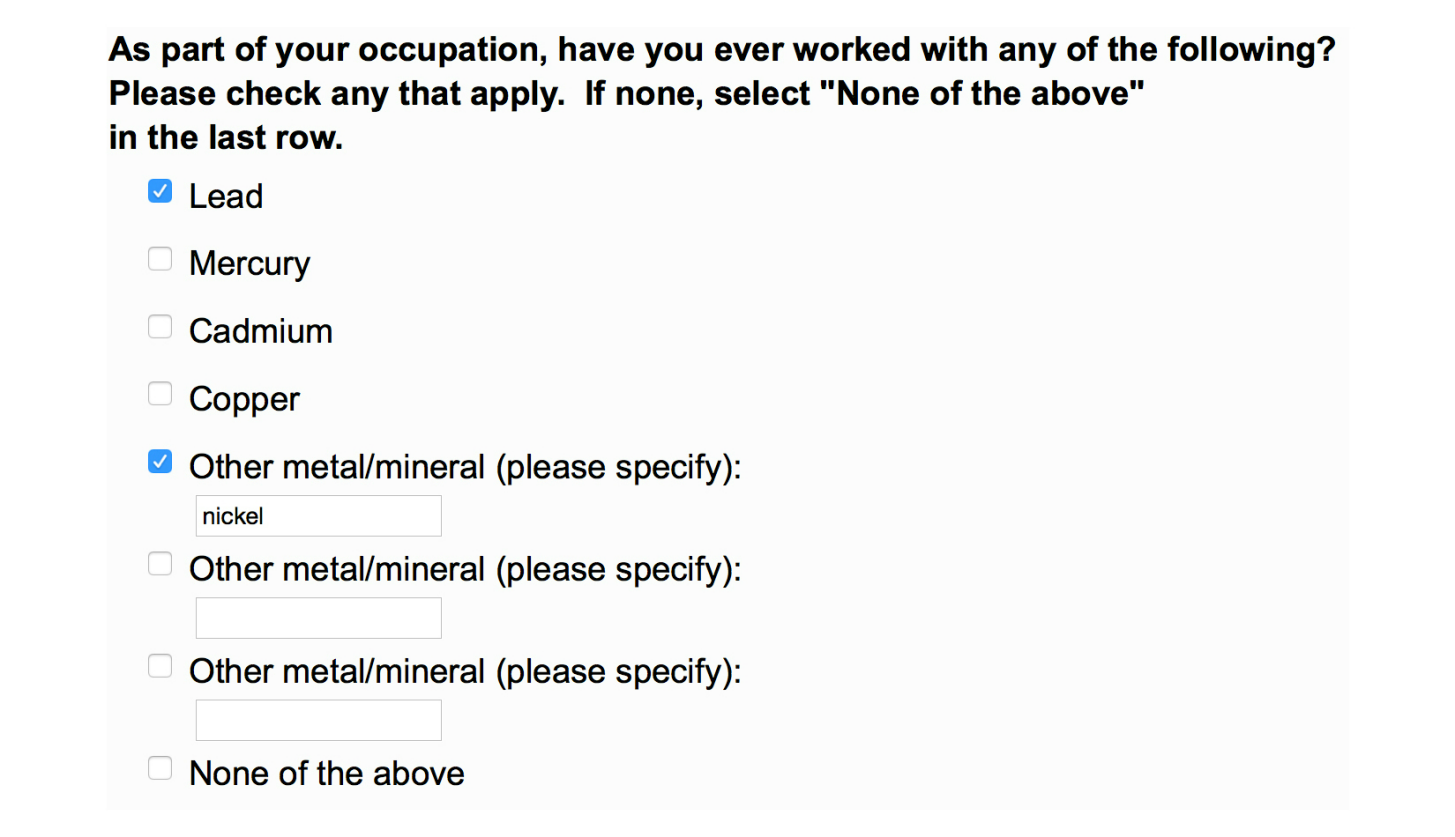
In an all-that-apply question respondents can tick as many answers as they want. In this particular question about occupational exposures there is a possible mix of tick-boxes and script entries.

**Figure 4:**
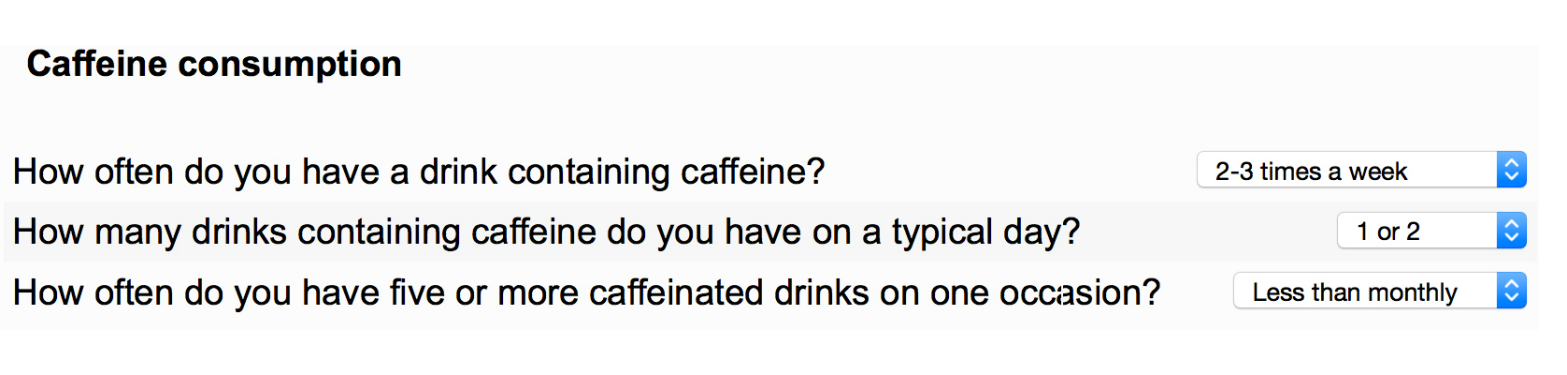
In these three questions about caffeine consumption respondents pick predetermined answers from drop-down lists.

**Figure 5:**
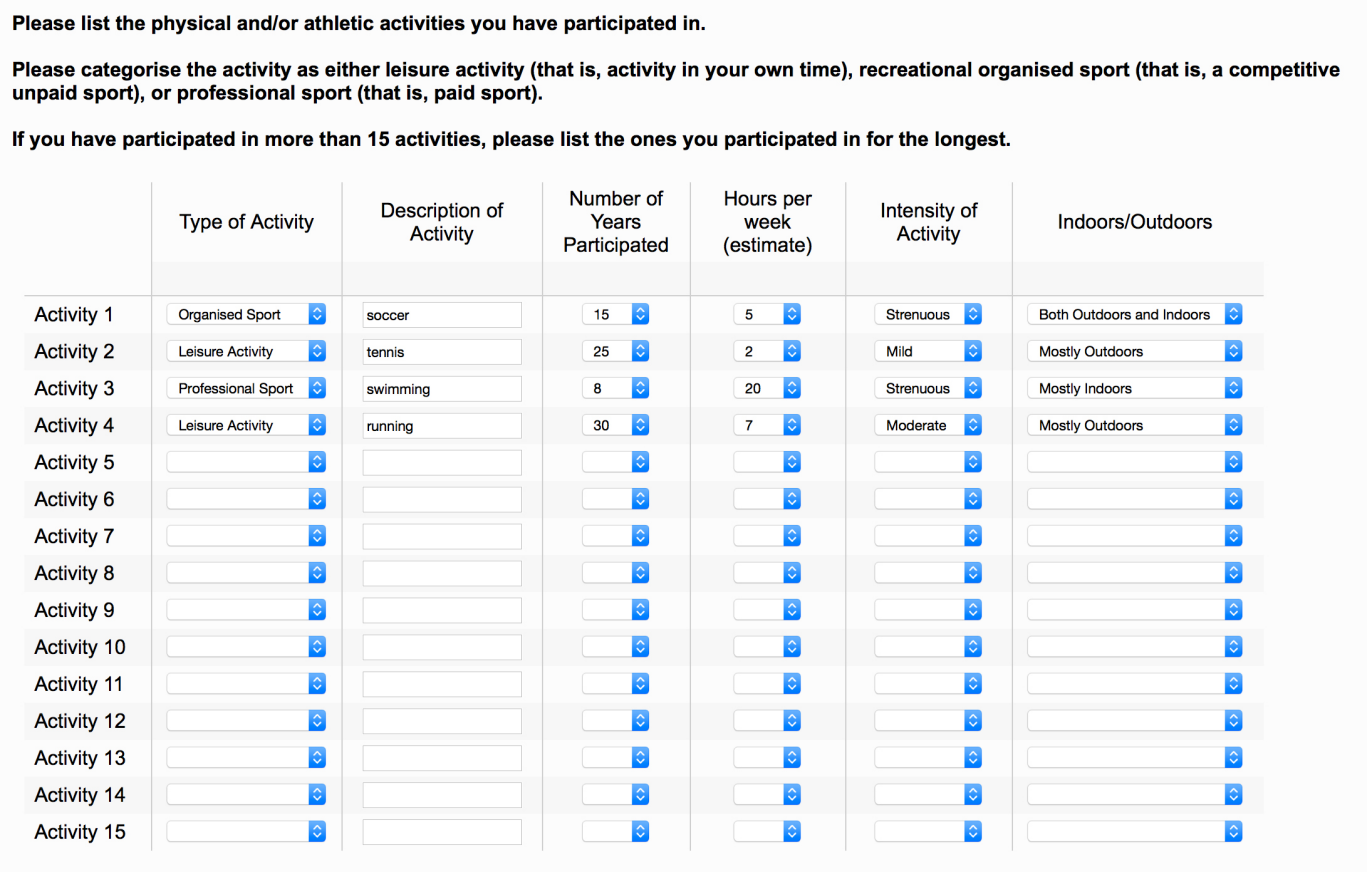
A large amount of information about the type, duration, intensity, and category of an activity can be obtained using side-by-side drop-down menus and script entry.

The questionnaire can be viewed online [16]. Examples of multiple choice questions are shown for Single Choice (Figure 2), Select All That Apply (Figure 3), and Drop-Down Menu (Figure 4) questions. An example of a Side-by-Side question is shown in Figure 5.

### Pilot Assessment

Ten people were asked to complete the questionnaire and provide feedback to test the clarity of the questions and the functionality of the questionnaire on multiple Internet browsers and devices. Based on this feedback, we adjusted some of the instructions for completing the questionnaire and edited the wording and format of some questions and choices of answers. In addition, after the survey first went online we received email feedback from some of the first 112 respondents. On the basis of this feedback a few minor changes were made and some questions were added. These changes did not affect the validity of the initial 112 responses.

### Acceptance and Initial Uptake of the Questionnaire

After approval from an institutional ethics committee, the questionnaire was placed online on 30 January 2015. Four months later, 379 responses (204 from ALS patients and 175 from controls) had been collected. In comparison, after 4 months we had received only 46 respondents from the same population using our paper-based questionnaire.

Spontaneous feedback via email; verbal feedback at meetings of ALS patients (including those with physical disabilities) and their partners; and comments from scientific and medical colleagues concerning the questionnaire format, its content, and ease of use have been positive. However, because we did not formally ask for this information from all respondents this feedback is not quantifiable.

Respondents report taking about two hours to complete the survey, and some appeared to complete it over multiple sessions. The majority of respondents so far have been from Australia since recruitment from countries outside Australia is in the initial stages. We will be promoting the non-English language versions of the questionnaire as their Google-translated versions are checked.

### Ages of Respondents in the Paper- and Web-Based Questionnaires

The average age of the first 379 respondents to the online questionnaire was 54 years (SD 15, range 18-86) compared to an average age of the first 379 respondents to the paper-based questionnaire of 60 years (SD 11, range 28-90).

## Discussion

###  Advantages of Web-Based Questionnaires in Neurodegenerative Diseases

Large numbers of responses can be acquired at low cost with minimal staff requirements and within a short period of time. This is especially relevant to some of the less common neurodegenerative disorders with short survival periods where traditional survey methods have had difficulty recruiting adequate numbers of respondents. Questions can be added easily when newly proposed risk factors are suggested. New risk factors for neurodegenerative diseases are continually being proposed, and with the advent of next generation DNA sequencing, the search for gene-environment interactions underlying these diseases is likely to accelerate. Automatic transfer of response data into database, spreadsheet, and statistics programs virtually eliminates the possibility of transcription errors and speeds up the data analysis. It also reduces the cost of running these surveys so they can be operated for longer periods, an important consideration when recruiting respondents with rare diseases. Other advantages of Web-based questionnaires have been well documented [45-49].

### Studies Comparing Online Versus Other Survey Modes

A review of 29 studies with a combined total of more than 15,000 respondents comparing different survey modes (postal mail, fax, email, and Web-based surveys) reported that Web-based surveys provided a better quality of response, greater level of detail, and greater compliance in answering open-ended questions than mail surveys [50]. The authors calculated similar response rates for the Web-based (52%) and mailed (51%) modes but found that average response times for Web surveys (7 days) were shorter than for mail (17 days). A population survey of 3148 Danish parents concerning their children’s health and welfare found similar response rates comparing paper, paper with Web option, Web-only, and Web with incentive formats [51].

The Black Women’s Health Study of 59,000 African-American women reported that Web-based surveys were filled out more completely than paper surveys and cost only 25% of paper surveys. Web-based response rates were greatest for younger age groups [52]. In the French NutriNet-Santé study of lifestyle and health, 94% of 147 volunteers stated a preference for the Web-based over the paper version [53]. Furthermore, this study found that the Web-based version prevented the omission of approximately 2% of answers (more than 550 values), which increased the value of each response. It also noted the cost benefits of the Web-based approach.

These studies demonstrate that Web-based surveys are as effective or better than other modes in garnering survey responses and obtaining sound data. These findings largely address the fundamental concerns of maintaining data validity and obtaining sufficient numbers of responses raised when making the decision to migrate to a Web-based platform.

### Online Surveys in Epidemiological Research

Despite results showing that Web-based questionnaires are as good or better than other survey modes, the field of epidemiological research has been slow to adopt Web-based methodology. A meta-analysis of epidemiology-related publications in seven high-impact general medical and epidemiological journals in 2008-2009 found that only 1% had used any form of Web-based data collection, while interviews were used in 28% and paper-based questionnaires in 29% (some used multiple formats) [45]. There is therefore potential for growth in the use of Web-based data collection tools for epidemiological purposes. The migration to Web-based questionnaires is likely to increase as a growing proportion of the population gains Web access. For example, World Bank data show that 83% of Australian and 46% of Chinese populations now use the Internet [54].

### Online Surveys in ALS Research

There appears to be only one other Web-based epidemiological study of ALS [44]. In that study, ALS patients who had enrolled electronically with the US National ALS Registry were recruited via email. Inclusion criteria were a diagnosis of ALS confirmed by a physician, knowledge of English, residence in the United States for at least 10 years, and age 21 years or older. Exclusion criteria were having also been diagnosed with Parkinson disease, parkinsonism, Alzheimer disease, dementia, poliomyelitis, or post-polio syndrome or having a family member with ALS. From the 2232 emails sent, completed surveys were received from 256 respondents who fulfilled the eligibility criteria, an enrollment rate of 11.5%. Among the topics covered in the survey were lifetime occupational history, occupational exposures, residential history, hobbies, physical activity, and military history.

We have also been given permission to recruit ALS patients from the US National ALS Registry. It will be of interest to compare our enrollment rate with that of Malek et al [44] since we have fewer exclusion criteria. Also, because our questionnaire is anonymous we predict more people will feel comfortable supplying personal information about themselves.

### Limitations of a Web-Based Questionnaire

#### Nonresponse Errors

A major concern in any survey is that the responses received are not representative of the population sampled (ie, nonresponse errors). It has been noted that the demographics of Internet users differ from the general population in that they tend to be younger and more educated [46,47]. However, one study that examined computer literacy and educational status among Web survey participants found that a substantial portion of their respondents considered themselves inexperienced in computer and Internet skills, and that those with less education were more accepting of the burden of completing an Internet survey [34].

A review of 11 Web-based surveys of people aged 65 years or older found that limitations for this age group were similar to those among all age groups [55]. One of the studies included in this review found that the mean age of Web-based participants (70 years) was lower than the age of face-to-face respondents (81 years) [56]. In our study, the average age of Web-based respondents (54 years) was slightly younger than that of paper-based respondents (60 years). This may imply some preference for the Web-based questionnaire among younger people, but direct comparisons between respondents in these two questionnaires are difficult to make. In our paper-based questionnaire, for example, all respondents also had to give a blood sample, which may have discouraged some younger people from participating.

Of note in our study, respondents are likely to be in the 40 to 70 year age group since this is the typical range for ALS. Therefore, age and educational status are unlikely to substantially limit participation in our Web-based questionnaire. We think that nonresponse error for our questionnaire will be minimal since most respondents are likely to have a strong interest in the subject.

#### Concerns About Safety of Personal Information

As with all uses of the Internet, there are concerns about safety and confidentiality of the data provided [45]. Our questionnaire largely circumvents this issue because all data are being collected as anonymous responses, and our data are secured on password-protected servers and computers.

#### Inability to Get Further Information or DNA Samples From Respondents

Since we have no identifying details on our respondents, we cannot contact them individually to ask them further questions or to ask for DNA samples to look for gene-environment interactions. However, there are now a number of databases containing large numbers of DNA samples from ALS patients, and should our study find risk factors for ALS, the same factors could be sought from patients who have donated DNA to these registries.

#### Inability to Obtain Physician Confirmation of Diagnosis

Since the responses are anonymous, we cannot obtain physician confirmation of the diagnosis of ALS or classify the cases using El Escorial criteria [57]. This is unlikely to be a major limitation since most ALS patients are well aware of their diagnosis. For example, of 88 people who self-reported a diagnosis of ALS to the US National ALS Registry, a check of their physician reports identified only 5 (6%) who did not have ALS [44]. We think the accuracy of the self-reporting of ALS diagnosis by our respondents will be improved by requesting them to select which subtype of ALS they have and asking them to contact their physician if they do not know this.

### Conclusions

The majority of epidemiological studies have been conducted using paper-based questionnaires, face-to-face interviews, or telephone surveys. The literature now shows that Web-based questionnaires offer many advantages over traditional methods with few drawbacks. Our experience creating an online questionnaire illustrates these advantages. Furthermore, our questionnaire is being translated into non-English languages and opened up to participation worldwide. We hope the data obtained from this project will accelerate our understanding of ALS and lead to the development of effective treatment options and preventative strategies.

## Appendix

Multimedia Appendix 1 The previous paper-based ALS risk factor questionnaire used by the Australian MND DNA Bank. A number of these questions were modified to fit the present online format.

## Abbreviations

ALS: amyotrophic lateral sclerosis
ALS-FRS: ALS Functional Rating Scale
FTD: frontotemporal dementia
MND: motor neuron disease
